# Supplementary material for: Smooth Interpolating Curves with Local Control and Monotone Alternating Curvature
Source: Comput Graph Forum. 2022 Oct 6;41(5):25–38. doi: 10.1111/cgf.14600 (PMC9827861; doi:10.1111/cgf.14600)
Supplement: Supplementary file 1 — Supplement Material [file CGF-41-25-s001.zip › Local-Smooth-Interpolating-MonoCurvature/extern/clothoids/submodules/quarticRootsFlocke/docs/api-cpp/namespace_PolynomialRoots.html]

Namespace PolynomialRoots — Quartic Roots v1.0.0

### Navigation

- index
- toc
- next
- previous
- Quartic Roots »
- C++ API »
- Namespace PolynomialRoots

# Namespace PolynomialRoots¶

Contents

- Detailed Description
- Classes
- Functions
- Typedefs

## Detailed Description¶

Implementation of Flocke algorithm for roots of 3rd and 4th degree polynomials.

There are 3 classed for 2nd, 3rd and 4th degree polynomial. An experimental translation to C++ of a C implementation of JenkinsTraub algorithm is available.

**References**

- **N.Flocke**

  Algorithm 954: An Accurate and Efficient Cubic and Quartic

  Equation Solver for Physical Applications

  ACM TOMS, vol 41, n.4, 2015
- **M.A. Jenkins and J.F.Traub**

  A Three-Stage Algorithm for Real Polynomials Using Quadratic
  Iteration

  SIAM Journal on Numerical Analysis

  Vol. 7, No. 4 (Dec., 1970), pp. 545-566

## Classes¶

- Class Cubic
- Class Quadratic
- Class Quartic

## Functions¶

- Function PolynomialRoots::roots

## Typedefs¶

- Typedef PolynomialRoots::complexType
- Typedef PolynomialRoots::indexType
- Typedef PolynomialRoots::valueType

### Quick search

### Table of Contents

- Quartic Roots Solver
- C++ API

«
hide menu

menu
sidebar
»

### Navigation

- index
- toc
- next
- previous
- Quartic Roots »
- C++ API »
- Namespace PolynomialRoots

© Copyright 2021, Enrico Bertolazzi.
Created using Sphinx 4.2.0.
